# Towards Integration of Environmental and Health Impact Assessments for Wild Capture Fishing and Farmed Fish with Particular Reference to Public Health and Occupational Health Dimensions

**DOI:** 10.3390/ijerph5040258

**Published:** 2008-12-08

**Authors:** Andrew Watterson, David Little, James A. Young, Kathleen Boyd, Ekram Azim, Francis Murray

**Affiliations:** 1 Occupational and Environmental Health Research Group, University of Stirling, Scotland; 2 Institute of Aquaculture, University of Stirling, Scotland; E-mails: dc11@stir.ac.uk (D. L.); f.j.murray@stir.ac.uk (F. M.); 3 Department of Marketing, University of Stirling, Scotland; E-mail: j.a.young@stir.ac.uk (J. A. Y.); 4 Public Health and Health Policy, University of Glasgow, Scotland; E-mail: k.boyd@clinmed.gla.ac.uk (K. B.); 5 Department of Physical and Environmental Sciences, University of Toronto, Canada; E-mail: eazim@utsc.utoronto.ca (E. A.)

**Keywords:** health impact assessments fishing aquaculture

## Abstract

The paper offers a review and commentary, with particular reference to the production of fish from wild capture fisheries and aquaculture, on neglected aspects of health impact assessments which are viewed by a range of international and national health bodies and development agencies as valuable and necessary project tools. Assessments sometimes include environmental health impact assessments but rarely include specific occupational health and safety impact assessments especially integrated into a wider public health assessment. This is in contrast to the extensive application of environmental impact assessments to fishing and the comparatively large body of research now generated on the public health effects of eating fish. The value of expanding and applying the broader assessments would be considerable because in 2004 the United Nations Food and Agriculture Organization reports there were 41,408,000 people in the total ‘fishing’ sector including 11,289,000 in aquaculture. The paper explores some of the complex interactions that occur with regard to fishing activities and proposes the wider adoption of health impact assessment tools in these neglected sectors through an integrated public health impact assessment tool.

## 1. Introduction

**Figure 1:**
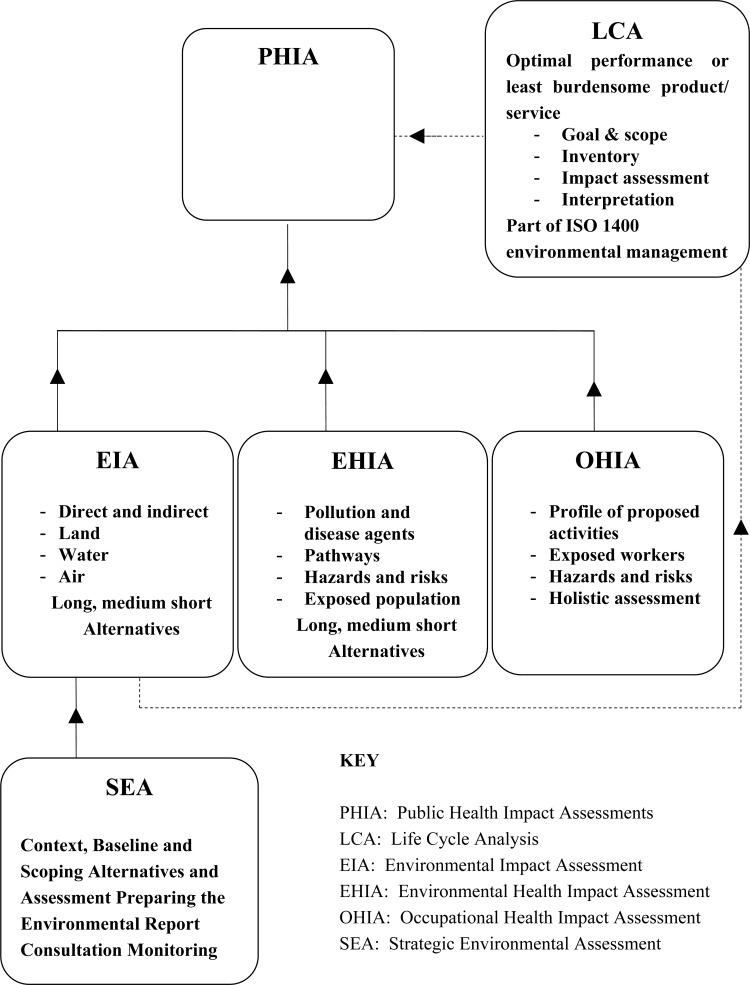
How impact assessments are inter-related and may be integrated.

Health impact assessments are viewed by a range of international and national health bodies and development agencies as valuable public health tools. Yet such tools have been generally ignored with regard to assessing impacts of both wild capture fisheries and rapidly expanding fish farming sectors. This is in contrast to the extensive application of environmental impact assessments in the same sectors. These sectors often operate in highly vulnerable, marginal, isolated and self-contained communities and in demographic and epidemiological terms that merit urgent attention to their health impacts. The numbers employed globally in fishing are enormous and increasing. In 1990, over 27,737,000 people were at work in the industry, of whom 3,832,000 worked in the ‘aquatic life cultivation sector’ [1]. The corresponding figures reported for 2004 were 41,408,000 people in the total sector and 11,289,000 in aquaculture. This paper proposes an extension of health impact assessment tools to these neglected but evolving sectors and the use of a new integrated public health impact assessment tool. It examines why a range of such assessments within environmental health, occupational health and under a public health impact assessment umbrella might be used and to what benefit in the two sectors. How these assessments may be integrated is illustrated below in figure 1.

Wild capture fisheries and fish farming provide a range of economic, social, psychological and physical benefits alongside a range of risks and costs to those who work in these industries, those who support and depend upon them and those who consume fish [2]. Fish farming, also described as aquaculture, which may include cultivation of aquatic plants such as seaweed, is a relatively recent growth sector of global food production since the 1970s and has since become one of rapid expansion and concentration which produced around 52 Mt in 2007; in 2008 it is anticipated that the contribution to global fish supplies for human consumption from aquaculture will equal that of capture fisheries [3]. Aquaculture production is dominated by China, which accounts for some two-thirds of global volume, with substantial shares of the remainder also from other Asian producers [1].

The EU, USA and Japan account for over 70% of global fish imports and this dislocation of production and consumption fuels a myriad of marketing channels to support international trade. Indeed in developing countries the net export value of fishery products (i.e. the total value of their exports less the total value of their imports) has grown significantly over the past 20 years and are substantially higher than for other agricultural commodities; being for example more than double the combined totals of coffee, meat and tea [3]. In such a dynamic and heterogeneous global market, environmental, environmental health and health impacts may potentially be considerable and, for a much smaller population, so may be occupational health and safety impacts. Assessment of these impacts should apply equally to wild fishing and fish farming.

Environmental impact assessments (EIAs) have been used with regard to fisheries from an early date [4]. The first formal EIA in the USA was undertaken in the early 1970’s and the first aquaculture application came in the same country in the early 1990’s followed by Ireland and Scotland shortly thereafter. The Bay of Bengal fisheries development program conducted one of the first environmental assessments for six countries in this key regional area 1991–1993 [5]. EIAs are used as a tool for assessing some of the effects of fish farming [6]. The sustainability of aquaculture too has been periodically addressed [7]. Environmental health impact assessments (EHIAs hereafter), drawing on the development of health impact assessments have been equally widely used for many decades by health and planning agencies [8]. However, EHIAs and reference to ‘public health’ have not formed any part of many such environmental impact assessments even where they do touch on fish farming and most do not [6, 9–12]. Similarly there have been scant references to human health in such analyses [13].

In 1997, an important appeal by E.S. Garrett was made to address these shortcomings in the USA and globally. “The aquaculture industry must have a better understanding of the impact of the “shrouded” public and animal health issues: technology ignorance, abuse, and neglect. Cross-pollination and cross-training of public health and aquaculture personnel in the effect of public health, animal health, and environmental health on aquaculture are also needed. Future aquaculture development programs require an integrated Gestalt public health approach to ensure that aquaculture does not cause unacceptable risks to public or environmental health and negate the potential economic and nutritional benefits of aquaculture” [14]. Yet the call does not appear to have been taken up in any systematic way.

The importance of such assessments in developing countries is enormous and may be complex [15]. For instance, wild capture fishing and fish farming in Africa may lead to greater prosperity among workers in these sectors than in agriculture [16] as well as supplying food needs [17]. With such activity may therefore come better public health. However, in some regions heavy fishing may damage health in terms of loss of an important food source. In Malawi, for example, where schistosomiasis is prevalent, heavy fishing can reduce snail-eating fish abundance and hence increase human health risks [18]. Yet in other parts of Africa such as Kenya, tilapia fish control mosquitoes and hence may help to reduce malaria cases [19]. Assessment of the environmental and health impacts of such activities can establish both beneficial and potentially damaging effects and side-effects which in turn will inform and aid decision-making regarding appropriate practice.

This paper therefore explores some of the existing literature on what appear to be disparate approaches to impact assessments and its relevance to fisheries. It then examines the merits of integrating such assessments under the umbrella of the public health impact assessment as a means to ensure the better evaluation of the health impacts of wild capture fishing and fish farming. Life Cycle Analysis (LCA) is beyond the scope of this paper but it should be noted that, under ISO 14040 on LCA, impact assessments form part of the LCA picture with respect to consumption of energy and materials in a production process. Linked to the sustainability of wild fisheries and the rapidly growing global fish farming sector, the integrated health assessments proposed in the paper should fill an important gap in practice and contribute to better public health policy development in the future.

## 2. Environmental Impact Assessments [EIAs]

“Environmental impact assessment” denotes the attempt to predict and assess the impact of development projects on the environment” [20]. Environmental impact assessments frequently examine the impact of human interventions on wild fish populations and as a matter of course may examine the health and public health impacts of such interventions on drinking water and fish consumption [21]. Hence EIAs will often automatically integrate an environmental health impact assessment component but, almost without exception, occupational health impacts as such are not addressed. EIAs’ scope has now widened from development projects in developing countries to projects proposed in developed countries and they are often now required under legislation in both the USA and Europe.

Strategic Environmental Assessment (SEA) is “a process to ensure that significant environmental effects arising from policies, plans and programmes are identified, assessed, mitigated, communicated to decision-makers, monitored and that opportunities for public involvement are provided”. Whereas EIAs tend to be very specific (to a project or activity), the SEA is “a generic and broader tool that has become an important instrument to help to achieve sustainable development in public planning and policy making” [22]. It is used to support sustainable development; to improve the evidence base for strategic decisions; to facilitate and respond to consultation with stakeholders; and to streamline other processes such as EIAs of individual development projects. SEAs now operate in the EU but are not a replacement for EIAs. They were introduced in Scotland and elsewhere in the UK in 2004. “The EU directive aims to provide a high level of protection to the environment and to contribute to the integration of environmental considerations into the preparation and adoption of plans and programmes with a view to promoting sustainable development, by ensuring that. . .a SEA is carried out on certain plans and programmes which are likely to have significant effects on the environment (European Parliament, 2001) [23]. Fisheries and agriculture are two of the sectors required to conduct SEAs.

## 3. Health Impact Assessments [HIAs]

Health impact assessments are “a combination of procedures, methods, and tools by which a policy, program, or project may be judged in terms of its potential effects on the health of a population, and the distribution of those effects within the population” [24]. Health impacts with regard to various forms of fishing and fish farming would require a wide assessment possibly where data may not be readily available. The impact assessment should relate to direct consumption of fish and their positive, negative, neutral and unknown effects as food. A comprehensive impact assessment might also include an investigation of behavioural impacts and the extent to which different socio-economic groups act upon health messages about wild and farmed fish. The indirect environmental health impacts in terms of sustainability (fuel, products, transport, packaging, disposal, energy and water use and so on linked to resource usage and public health effects), pollution, contamination, food handling would also require assessment; as would occupational health impacts at different levels of the marketing chain. However, tools for such assessments may not be readily available or easily applied.

Such an approach would provide a valuable health evidence base for either introducing, maintaining and developing various types of fishing and fish farming or perhaps not developing them at all. Yet very little if any classical coherent and organised HIA screening, scoping, policy analysis, profiling, qualitative data collection, impact analysis, prioritising impacts or process evaluation and monitoring of impacts and outcomes [25] has been done on aquatic production. Whereas the principles of HIA that might apply to fishing have been outlined, fishing itself has not been addressed in general texts [26] although they merited early discussion with regard to development projects [1, 27].

International Health Impact Assessments, where carefully evaluated, would shed useful comparative light on wild capture fisheries and fish farming. “Sustainable development with its social objectives of empowerment, participation, equity, poverty alleviation, social cohesion, population stability and institutional development is an appropriate framework for conducting health impact assessments” [28]. If applied as a regulatory process and with full transparency and rigour, it may help to engender greater public confidence in the technique as a means of resolving some conflicts over data and evidence used to assess consumer and wider health impacts. There may be significant benefits too for environmental justice and community involvement in HIAs [24, 29].

What does exist is a large and rapidly growing literature on the benefits and risks of fish consumption and the trade-offs that may occur between these [30] - in terms of both nutrition and possible chemical contaminants for instance - as well as the proliferation of certification schemes and ecolabels linked to a trend for regulation of social and environment impacts by market forces [31]. However, it is not the purpose of this paper to review that literature rather to link the literature, where appropriate and possible, to the field of health impact assessment. Efforts have been made to combine studies of both potential health benefits and adverse effects of fish consumption through the use of quality-adjusted life years or QALYs [32] which is a generic measure of health benefit incorporating improvements in survival with quality of life. Additionally, recent important studies have specifically begun to analyse the impacts of risk communication on fish consumers [33, 34] and other consumer perceptions of health linked to particular fish species [35].

The specific health impacts of eating fish have not yet been fully resolved with a wide variety of claims made - some with a stronger evidence base than others – for improved survival, wider health benefits and also for detrimental effects for consumption of certain species. Definitive guidance for consumers may be unclear. Impacts may also vary considerably for some groups depending on location, type of fish consumed, amount and frequency of fish consumed, levels of contamination present and impact on total body burden of that contaminant and combinations with other contaminants, and age, lifecycle stage and gender of consumer. For instance there is a scientific consensus that pregnant women should limit but not end their oily fish consumption, tailoring it to specific ‘low mercury’ species. There are also suggestions that exposure to substances such as polychlorinated biphenyls (PCBs) in contaminated sports fisheries in North America and in fish in European waters may have had an impact on sex ratios [36, 37] and other studies that indicate effects on birth weights of the offspring of women consuming fish in the same area are unaffected by contaminants [38].

### 3.1. Health Benefits –Wild fish and Farmed fish

‘Health benefits’ may consist of eating fish that contribute to good health or possibly as a substitute food that does not contribute to ill-health for alternatives which do. There is now a considerable literature and much debate about the relative benefits versus risks of fish consumption. “Fish are important sources for many nutrients, including protein of very high quality, retinol (Vitamin A), Vitamin D, Vitamin E, iodine and selenium. Evidence is increasing that the consumption of fish enhances brain development and learning in children, protects vision and eye health, and offers protection from cardiovascular disease and some cancers. The fats and fatty acids in fish, particularly the long chain *n*-3 fatty acids (*n*-3 PUFA), are highly beneficial and difficult to obtain from other food sources” [11]. Some evidence is also emerging about links between fish diets and behaviour [39].

What wild fish eat cannot be controlled and hence there are concerns about the potential adverse health effects of heavy metal contamination of such fish from both natural and industrial sources. Farmed fish may be fed with feed that can also be contaminated for example with wild sourced fish meals and oils or other feeds and hence their health impacts too could be negative [40]. However, if the farmed fish feed is not contaminated with heavy metals and other pollutants, their potential adverse health effects would be minimal. These types of impacts are for a variety of reasons rarely fully assessed or reported yet would be highly relevant given the consumer and media interest in food contamination.

### 3.2. Health Risks

Most concerns about the health risk posed by fish generally relate to contamination and concentration of heavy metals especially mercury. Also of particular concern are chemicals such as PCBs and some persistent organic compounds from agriculture and industry as well as naturally occurring toxic metals and chemicals [41]. Evidence exists of oestrogenic and androgenic endocrine disruptors affecting marine as well as freshwater species – with human health implications [42]. There could also be health risks from fishborne zoonotic parasites which may be a serious threat to health in developing countries but are a minimal threat in developed countries where many problems relate to over-consumption of certain foods. Food hygiene and food storage practices may create a more significant risk to consumers from food poisoning. Recent studies have also demonstrated the difficulty of assessing risks and benefits of fish accurately because of the presence of many confounders [43]. However, integrated health impact assessments of these potential risks are rare if not unknown, in contrast to the many cost-benefit and risk-benefit health analyses of fish consumption. Nor did any of the studies on health risks and benefits explore the indirect health impacts of fish production, processing and transport.

## 4. Environmental Health Impact Assessments [EHIAs]

Where an environmental impact assessment explores human health, “this is often called an “environmental health impact assessment.” It is widely held that such impact assessment offers unique opportunities for the protection and promotion of human health” [20].

Assessing impacts of environmental health policy can be highly problematic although increasing efforts are being made to integrate various forms of risk analysis and cost-benefit analysis in environmental health [44].

With regard to all forms of fishing/ fish farming, an EHIA or a full life cycle analysis that included an EHIA would need to include the use of resources and impacts on environmental health. This would include for instance environmental health impacts on air, water and land ranging from:

- extraction of raw materials or feeds (including fish as feed) needed for development of equipment, machinery and materials for keeping or catching fish
- energy, water and land requirements for farming fish
- energy and material requirements for catching fish
- the high perishability of fish as a raw material compared to other foods and consequential limited time for processing/transformation
- impacts for chilled/cold chains and potential for energy-intensive processing/distribution
- operating factories and warehouses related to fish processing
- disposal of fish and fish products
- transporting fish and processed and packaged fish [45]

Many of these matters would be closely assessed in financial terms by businesses but EHIAs prior to the commencement of such activities would be rare if not unknown. For instance fish is commonly sold as a fillet or otherwise prepared product rather than in its live weight format. Such preparation typically results in yields of no more than 50%, a loss which may encourage processors to seek out sources of cheap skilled labour. Consequently fish may be transported from the point of production to some distant location for processing before being returned for sale. In other instances the limited shelf life of fresh fish may necessitate air transport rather than other modes of distribution [45, 46]. Yet the impacts of the complexity of these channels have received only scant attention. For example the contribution of shipping to oil usage and air pollution as a whole and hence to global warming with related adverse health impacts has been neglected until very recently.

Yet there will be large sectoral and other differences operating. For example the environmental health impacts of locally caught wild fish may be minimal in terms of pollution and the lack of fish feed production and waste contamination, anti-fouling agents [47, 48].

A potential merit of aquaculture is the ability to locate different production systems, to varying extents, with respect to markets and/or resource inputs. Recirculating Aquaculture Systems (RAS), the most production and energy intensive system-type, also has the greatest flexibility in this respect. Thus the transport costs and fish miles of such farmed fish may be very small and marginal energy consumption on fish farms that draw on other sources of energy already being generated may be small. However, such an assessment would be very speculative and inconsistent with available LCA results [49–51]. Nevertheless more than 55% of aquaculture production now enters global value chains so ‘fish miles’, like other ‘food miles’ will not be negligible. These largely industrial operations also rely on imported feed ingredients to varying degrees and it is the environmental services including water and land that are likely to be the primary determinants of site location. Environmental impacts may vary too between species: for example the most mature industrial sectors for salmonids in temperate water require more than 3 years grow-out compared to less than one year for most tropical finfish. These anadromous species also impact on both fresh water and marine environments, incur high live-fish movement costs and are top end predators with high quality dietary requirements [52]. The impacts of greenhouse nitrogen species (N, N20 and NH3) from RAS are potentially high but require further research [48]. In a land-based turbot farm in Norway using waste heat, feed production had the greatest environmental impact [53].

In the Danish flatfish fishery, the fishing stage has the largest impact potential for the investigated impact categories. An LCA of this fishery found the fishing stage had the greatest environmental impact (fuel, biocide and fouling agent emissions) – similar for many other fish species. Herring, mackerel and mussels were exceptions where the processing phase was more environmentally significant due to energy intensive packing [47, 54].

Hence air pollution and contributions to global warming may be much smaller than might at first appear to be the case. Nevertheless there are many practical aspects determining the spatial distribution of aquaculture which tend to countermand these theoretically possible benefits. Fish trade has been recognised to be dominated by the growth of global value chains, encouraged by increasingly concentrated transnational organisations, whose logistics and attendant supply chains do not always ensure low fish miles, or low impact upon resources such as land and water. Moreover the species that have been favoured historically by such organisations, notably salmonids, have characteristics which are less environmentally friendly than alternative options.

Very occasionally EHIA statements refer to hazards to workers and their families [8, 55]. However, these tend to be somewhat tangential and incomplete areas of activity with occupational health and safety generally overlooked in the practical application of EHIAs.

## 5. Occupational Health Impact Assessments [OHIAs]

Work environments generally tend to be the Cinderellas of the public health world [56]. They are often ignored or marginalised within the wider field of public health. This may relate partly to the diminishing role of occupational health in government departments in many over-developed countries and to the propensity of civil servants working in the field to downplay the importance of the subject [57]. Yet work environments affect health and health affects the work that employees may do. Where health impact assessment papers have been published in the occupational and environmental health journals, they surprisingly do not directly relate the method to any aspect of occupational health, perhaps because of the current weakness of occupational health as a discipline, although they do so for environmental health [58].

An assessment of the health and safety impacts of various types of fishing/fish farming would properly include such things as the machinery and equipment used, drowning risks, boats and buildings, systems of work, work organisation including shift work and night work, ergonomics including musculo-skeletal, repetitive strain and standing hazards, noise and vibration, lighting, personal protective equipment, exposures to heat and cold, knives and processing plant machinery, chemical exposures including dust, fumes and gases, zoonoses, confined space working, electrical and fire hazards and psycho social factors. No systematic assessments of all these elements and how they combine have been published in the literature [59, 60]. Fish processing hazards have attracted more attention for example in Africa and also in Scandinavia [61–63].

The hazards, primarily safety but also some occupational health elements, of some types of commercial pelagic fishing have been flagged for several decades, although the industry continues to have one of the worst global safety records in terms of fatalities [59]. Elsewhere, for example, crab fishing in the Baring Sea is viewed as a financially lucrative but highly hazardous occupation. The activity is determined by high market prices for catches linked to constraints from seasonal permits to conserve stocks with inevitable trade-offs not only on catches and the biological resource but also health and safety which is supported by various types of regulation primarily related to safety of the boats. Whereas safety has been explored in crab fishing [64], work on the health impacts of long hours, night work, catch-based payment systems and poor, sometimes hazardous, working conditions of the group are relatively under-explored.

Evidence indicates that lay groups and some risk professionals may seriously under-estimate or underplay the risks associated with commercial fishing [65]. Relatively little research exists on health and health-related problems facing fisheries and fish workers [66, 67]. None exists for fish farming. Yet large, and increasing, numbers of workers are reported to be involved in the activity [5]. In Chile for example, there were 850 aquaculture centres in 1999 that harvested over 300,000 tons of fish [68].

The International Labour Organization brings together employers, workers and national governments to address a range of workplace problems sometimes through conventions and agreements and sometimes through voluntary tripartite action. The ILO has produced a number of reports and held a variety of conferences dealing with fishing [69, 70]. Fish farming has been briefly touched upon in a number of these events but the main foci have been on whether fish farming was an agricultural or fisheries sectoral interest and to what extent fish farming could contribute to solving food insecurity in developing regions and related problems of poverty. These concerns often over-ride but do not entirely swamp concerns about the health and safety of fish farmers in such countries as Pakistan [71]. Health and safety may also be neglected in existing and emerging certification standards. No attempts have yet been made by international bodies such as the FAO or ILO to assess in any comprehensive way the ‘public health impacts’ of fishing and fish farming that builds in a proper assessment of occupational health and safety related to other risks and benefits.

There will be major differences in any occupational health impact assessment between large-scale commercial fishing activities and small scale subsistence fishing. Although cultural attitudes to safety and risk may be similar between different fishing activities, the reasons for those attitudes will vary enormously: the former may be relatively highly paid for the risks they take but the latter may take risks to ensure food security for themselves and their families with few other choices. There are issues too about the uncertainty of employment in fisheries & aquaculture and also the ongoing tendency of capital to be substituted for labour and the low quality of many of the jobs available in the sector.

Fish farming, when compared with wild capture fishing and its occupational health and safety hazards has been neglected in the scientific literature with the exception of a pioneering paper on the farmed salmon industry [72], one review of potential hazards in the industry and one US research project currently underway [73, 74]. However, there may be greater similarities in terms of the risks faced by the two sectors with the likely future trend of some fish farms being located further out to sea and thus contending with even more dangerous working environments. Since the work of Douglas [72] some attention has been given to the hazards of fish farming in the context of safety and also the use of pesticides and other agents to control fish parasites or influence fish growth, size and colour. This has resulted in the banning or restriction of various hazardous chemo-therapeutants: for example anti-sea-lice products such as organophosphates, hydrogen peroxide and malachite green, now banned as a carcinogen [75, 76].

Regulatory information is even less readily available. Few occupational health and safety regulatory and enforcement bodies internationally use impact assessments although they will assess economic impacts and burdens of health and safety regulation. Most bodies simply incorporate fish farming within existing laws and codes of practice. The Norwegian occupational health and safety regulators produce reports on fish farm health and safety and use land based and maritime legislation to control the activity [77]. The US appears to have done more research and produced better information on the subject than any other country [59, 60]. Within the UK, the enforcement body, the Health and Safety Executive has produced leaflets on floating platforms in the fish farming industry but has no specific information leaflets on occupational health in the wider fish industry. It also established, with partners, a joint health and safety committee under the Maritime and Coastguard Agency umbrella. Canadian provinces, with significant numbers of fish farming enterprises, appear to have neither specific information on the subject nor any organisational structure for pursuing the matter. This contrasts with legislation for such sectors as mining, construction and agriculture generally.

This would seem to indicate that globally there is a lack of information available to assess health and work impacts of fish farming. Unionised labour often press for risk and other assessments in workplaces and can be sufficiently well organised to monitor the health impacts of fish farming. But often, because of labour market factors such as relatively small numbers of workers in the units, concerns over the limited availability of alternative employment especially in rural and remote areas, *inter alia*, fish farms are commonly not organised and may well use self-employed labour. There is also often a longstanding socio-cultural tradition of tolerating adverse working conditions within the localised fishing communities. The Maritime Union of Australia has organised some workers but that is the exception and not the rule [78]. Anecdotal evidence exists that a not uncommon practice in vertically integrated salmon companies is to transfer farm workers with back-injuries, (due to net-lifting) to less physically demanding jobs, for example in hatcheries or processing and thus avoiding industrial injuries claims against employers.

With limited or no data available, and with no statutory requirement to carry out health impact assessments in the workplaces, none exist. The assumption by regulators and industry may be that the risk assessments, hazardous substances assessments, safety (sic) policies and various regulatory packages ensure old and new workplaces deal effectively with health impacts of work. That this is not so is demonstrated by the global statistics on occupational diseases which reveal international failings. When new industries such as fish farming emerge in some countries or when ‘old’ industries such as fish farming appear in new countries or new areas, there are arguments for calling for OHIAs of those industries and their integration into wider public health impact assessments. If this is done, there will be clear links established between environment, health and work in a way that provides a fuller and more holistic picture of how the parts fit together and affect the world we live in.

There is an emphasis on victim blaming and psychological profiling in this field linked to risk perceptions rather than structural, work organisation systems, and the economic and political factors that shape the risks presented by various hazards. An impact assessment approach would serve to correct such skewing and, with regard to fishing and fish farming as well as other sectors, would provide a more rigorous basis for occupational health and safety activity.

It is a truism that ‘good work’ is good for your mental and physical health and ‘bad work’ may be bad for your mental and physical health. An OHIA of aquatic food production systems fishing could make a valuable contribution to the identification of good and bad working practices and the factors that come together to create both good and bad work.

## 6. Public Health Impact Assessments [PHIAs]

Public health agencies and public health professionals use HIAs. In this respect, it may be argued that PHIAs occur as a matter of course. Some useful examples do exist of efforts to explore the public health impacts of aquaculture primarily linked to food safety and drawing on hazard analysis and critical control point approaches [79]. However, this paper specifically proposes bringing together cognate EIAs with EHIAs together with the new concept of OHIAs (rather than narrow risk assessment tools for a specific process or material) and integrating them. In terms of information required and skills needed to perform such PHIAs, many of these will exist already within a range of agencies and draw on professionals already working in those agencies who have the relevant competencies. Alternatively, it may be possible to bring the missing expertise from outside into a PHIA team. Such PHIAs may therefore offer the means of integrating environmental, environmental health and occupational health impacts in ways that recognise all the major complex factors influencing public health. LCAs do not currently address either all the key elements of an EIA or EHIA and most ignore OHIAs.

It should be possible to carry out PHIAs without great extra cost or time burdens. Economic impacts of proposed projects are frequently assessed and again PHIAs will be able to draw on and utilise these without additional financial burdens being created. Data about employment will provide data about occupations and materials and hence about likely risks and hazards to the work and wider environment. The importance of public health in aquaculture has been recognised primarily in terms of employment and nutrition [80] but not all health impacts have been assessed. There may be additional large or small impacts on housing, social services, education, land usage, construction activity, health service provision and community viability in terms of population retention. Indeed the whole area of the somewhat mis-termed ‘social capital’ is better described as social or community resources and capacity.

Other ‘fishing industry’ factors that will impact on public health relate to such elements as the balance between subsistence and cash ‘food’ production. For instance commercial shrimp farms may potentially provide local communities with income and employment which can be positive but they may also damage the water supply and other subsistence aquaculture and agricultural activity with adverse effects in terms of pollution and other jobs [81]. The scale of a fishing activity may determine positive and negative public health effects. National public health may benefit from widely available fresh fish species supplied to local markets with low fish miles and hence low energy and pollution costs from transport. However, if markets of vulnerable communities in developing countries who produced fish for commercial sale were affected by such developments, this could have an adverse public health effect on these communities unless alternative markets or subsistence fish or other food production emerged [82].

Aquaculture may also affect public health in terms of the effects of fish feeds and fish veterinary products. The influence of global and regional trade agreements on products available may be considerable [83, 84]. Ongoing debate surrounds the scale and application of tariffs to fish products traded which in turn impacts upon the sustainability of the export networks supported. Similarly, the historical subsidisation of fish production has further complicated the contexture of economic systems and sustainability measurements [85]. This indicates that global as well as national and sectoral public health impact assessments of fishing may have their place and should be used to monitor the work of bodies such as the World Bank and the World Trade Organisation and the impact of their economic and funding decisions on fishing and fish farming activities more widely.

## 7. Conclusions and Recommendations

A variety of tools exist for assessing a range of hazards, risks and beneficial and adverse impacts to public health in the fishing and fish farming industry. Public health impact assessments proposed in this paper offer one of the most comprehensive and under-utilised tools available and will demonstrate best practice in the industry. They can bring together all the key relevant and necessary aspects of several types of impact assessment needed for a full assessment of an activity. Their dissemination and use should lead on to more effective and evidence-based hazard and risk management decisions at a range of policy levels [86]. This in turn should lead to better decisions which engage all parties - fish producers, fish sellers, fish consumers and fish workers – and inform better policies and practices. This will then contribute to the public health of communities, workers and consumers by adequate consideration of all the direct and indirect health and safety benefits, risks and costs of fishing activities.

As many of the different types of impact assessment discussed in the paper are already required, the time and cost implications of integrating them would not be great. It should also be possible to draw on necessary and existing data that are required for risk assessments and risk management of occupational health and safety and reframe and focus that data for a proper OHIA to add to the PHIA. Hence, within many development projects and in many settings within the northern hemisphere, integrated PHIAs should be and could be practical, valuable and feasible tools in the same way that health impact assessments already are. However, occupational health and safety assessments even in some established fishing and fish farming sectors are either poor or non-existent and the development of PHIAs that integrate occupational health will present a major challenge. In addition, the majority of fish farming in developing countries operate now without such assessments and the challenges here of using the PHIA tool may thus be great.

The ILO and FAO are critically important in facilitating such new developments and building on their past work in these industries [87] especially in developing countries and could take forward the PHIA approach proposed. The ILO produced a charter on the maritime industry in 2006 whilst much earlier the FAO produced its voluntary Code of Conduct for Responsible Fisheries, including fish farming in 1995. The Code encourages port States to check fishing vessels for compliance with subregional, regional or global conservation and management measures or with internationally agreed minimum standards for the prevention of pollution and for safety, health and conditions of work on board fishing vessels. The FAO Ministerial Meeting on Fisheries (Rome, March 1999) adopted the Rome Declaration on the Implementation of the Code of Conduct for Responsible Fisheries.

Hence PHIAs could provide excellent tools for providing early interventions in assessing health impacts of new schemes and should contribute to specific assessments of compliance with the various ILO and FAO codes and charters relating to fishing activities. They may be less effective but still of value in assessing existing fishing and fish farming activities. Moreover, they should be invaluable tools for use by workers in bodies such as the International Transport Workers Federation (ITF) or the International Union of Food, Agricultural, Hotel, Restaurant, Catering, Tobacco and Allied Workers’ Associations (IUF) who either organise fishers or who may have contact with workers on fish farms globally.

PHIAs may also be important tools that could be used by organisations wishing to assess the corporate responsibility of companies and investors active in fishing and fish farming sectors with regard to public health matters. Currently many companies that sell fish are seemingly more concerned about sustainability and consumer perceptions of ‘healthy’ fish stocks, and less involved with public health considerations that include worker and community health and safety. In this context, there could be additional opportunities to build in checks on public health, using PHIAs through certification schemes on various types of fish production.

The scope for such extension of the more holistic coverage of certification schemes is considerable and timely because ecolabel and certification schemes are becoming an increasingly important part of the international market for fish [88]. Some organisations have made claims about their strategic goals to market only fish certified according to certain approved standards [89]. Whilst this undoubtedly can be seen as a potentially significant barrier to entry for some producers, it might also be viewed as an opportune window to ensure incorporation of criteria no less pertinent to the future health of the fisheries and fish farming sectors. The scope for such inclusion of course presents a further set of challenges to reach the ethical decision-making consumer, but these might be considered to be small in relation to the tasks that incorporation of PHIAs currently encounter.
